# Small RNA Functions as a Trafficking Effector in Plant Immunity

**DOI:** 10.3390/ijms20112816

**Published:** 2019-06-09

**Authors:** Chen Zhu, Ting Liu, Ya-Nan Chang, Cheng-Guo Duan

**Affiliations:** 1 Shanghai Center for Plant Stress Biology and Center of Excellence for Molecular Plant Sciences, Chinese Academy of Sciences, Shanghai 201602, China; zhuchen@psc.ac.cn (C.Z.); tingliu@psc.ac.cn (T.L.); ynchang@psc.ac.cn (Y.-N.C.); 2 The University of Chinese Academy of Sciences, Beijing 100049, China

**Keywords:** Small RNAs, Cross-kingdom RNA silencing, Host-induced gene silencing (HIGS)

## Abstract

Small RNAs represent a class of small but powerful agents that regulate development and abiotic and biotic stress responses during plant adaptation to a constantly challenging environment. Previous findings have revealed the important roles of small RNAs in diverse cellular processes. The recent discovery of bidirectional trafficking of small RNAs between different kingdoms has raised many interesting questions. The subsequent demonstration of exosome-mediated small RNA export provided a possible tool for further investigating how plants use small RNAs as a weapon during the arms race between plant hosts and pathogens. This review will focus on discussing the roles of small RNAs in plant immunity in terms of three aspects: the biogenesis of extracellular small RNAs and the transportation and trafficking small RNA-mediated gene silencing in pathogens.

## 1. Introduction

Small RNAs (sRNAs) in plants with different lengths (21–24 nucleotide, nt) can be divided into two major groups: microRNAs (miRNAs) and small interfering RNAs (siRNAs), which are processed from a single-stranded hairpin RNA precursor or double-stranded RNA (dsRNA), respectively [1,2,3]. Since the discovery that dsRNA can trigger gene silencing in *Caenorhabditis elegans* [4], sRNA has become commonly recognized as an important signaling molecule in the regulation of plant development and abiotic and biotic stress responses through transcriptional gene silencing (TGS) or posttranscriptional gene silencing (PTGS) [5,6,7]. The machinery for RNA silencing (also known as RNA interference, RNAi) in plants consists of three core components: RNA-dependent RNA polymerases (RDRs), which are responsible for catalyzing the biosynthesis of dsRNAs from a single-strand RNA (ssRNA) template; DICER-LIKE (DCL) proteins, which cleave dsRNA or single-stranded hairpin RNA into sRNAs; and Argonaute (AGO) proteins, which are guided by sRNAs and bind to target mRNAs in a sequence-complementary manner, leading to mRNA cleavage or translation inhibition [8,9,10,11].

To survive and propagate in a challenging environment, plants have to cope with different kinds of pathogens, such as bacteria, fungi, viruses, and parasites. The pathogen-associated molecular pattern (PAMP)-induced basal resistance response in plants is referred to as pattern-triggered immunity (PTI) [12]. Plant intracellular nucleotide-binding/leucine-rich-repeat (NLR) receptors and the resistance protein (R protein) can detect pathogen effectors and induce a robust resistance response known as effector-triggered immunity (ETI) [13,14,15,16]. Effectors are small cysteine-rich proteins with signal peptides that are secreted by pathogens during host colonization to modulate host defense responses, thereby establishing host colonization [17]. The arms race between the host and pathogen is fierce because of the rapid evolution of effectors and R protein-encoding genes in different pathogen and host species [16]. Flor’s gene-for-gene theory indicates that a certain effector (such as a small secreted cysteine peptide or avirulence protein)-encoding gene recognized by the host R protein-encoding gene triggers ETI, which explains why only certain host species with R genes show resistance to certain pathogen races with effector-encoding genes [18].

Additionally, different sRNAs play essential roles in plant immunity during the perception of invasion by different biotrophic or necrotrophic pathogens. The DCL-dependent production of virus-derived siRNAs (vsiRNAs) and amplification of secondary vsiRNAs by RDRs are important during plant-virus interactions [19]. miRNA-mediated secondary siRNA production is also crucial for plant anti-viral immunity through R gene homeostasis [20]. In addition, the plant RNA-directed DNA methylation (RdDM) pathway, which establishes DNA cytosine CHH (where H is any nucleotide but G) methylation at 21-nt- and 24-nt long noncoding RNA targeting sites, is also involved in conferring plant resistance to DNA viruses [19]. The plant anti-bacterial defense conferred by siRNAs is largely dependent upon differences in the perception of bacterial effectors [21]. In addition, different siRNAs are induced by different effectors, even within the same bacterial species [21]. Several miRNAs are also upregulated after plants are infected by fungi such as *Verticillium dahlia* (a vascular invasive fungus) or *Botrytis cinerea* (causing agent of Gray mold) [22,23]. Partial target genes of these miRNAs have been identified to be essential for plant immunity [22,23].

In addition to the role of innate sRNA synthesis in conferring plant immunity, the mechanism of cross-kingdom RNAi has been identified in plant-pathogen interactions [24,25]. Recently, increasing evidence has indicated that sRNAs are capable of functioning as trafficking effectors during plant-pathogen interaction [26,27]. However, many scientific questions underlying this mechanism remain to be elucidated. How is sRNA biogenesis initiated when a plant confronts pathogens? How does a plant transport these sRNAs to pathogen tissues? How do the plant sRNAs function in pathogen cells? These questions will be further discussed in this review. 

## 2. Biosynthesis of sRNA Induced by Pathogen Invasion

### 2.1. sRNAs in Plant-Virus Interaction

**Figure 1:**
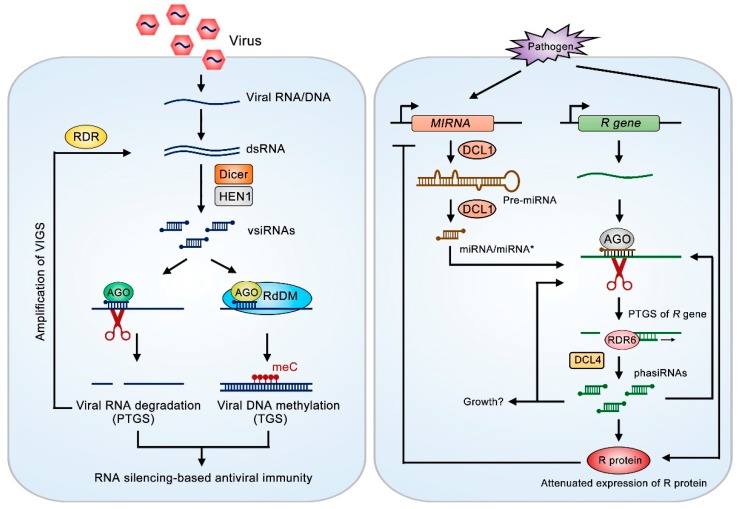
RNA silencing-based immunity and its cross-talk with *R* gene-mediated immunity. Left panel: RNA silencing-based immunity. Replication of viral RNA triggers the synthesis of dsRNAs, which are then processed by Dicer RNase into 21–24 nt siRNAs. Some 21–22 nt vsiRNAs are loaded onto Argonaute-RNA-induced silencing complex (AGO-RISC) complex (such as AGO1 in Arabidopsis) to mediate sequence-specific degradation of viral RNAs (PTGS). Virus-induced gene silencing (VIGS) can be amplified by RDR-dependent formation of dsRNA from the cleaved target mRNA. 24 nt vsiRNAs are loaded onto AGO-RdDM silencing complex to target viral DNA for DNA methylation modification, thereby leading to silencing of viral genes at TGS level. Some pathogens are capable of interfering with miRNA biogenesis to facilitate infection. Right panel: In plants, miRNA is firstly transcribed from *MIRNA* gene by pol II and processed into pre-miRNA, the stem loop precursor, and then processed into miRNA/miRNA* duplex by DCL1. Mature miRNA is loaded into AGO-RISC complex to trigger degradation of R gene mRNA, leading to attenuated expression of R protein. Some R gene-targeting miRNAs is capable of triggering the production of RDR6 and DCL3-dependent phased secondary siRNA (phasiRNAs) from the cleavage site of R gene mRNA. The R gene-derived phasiRNA can induce trans-acting silencing of R gene or other target genes (such as growth-related gene). In addition, perception of pathogen infection will activate the expression of R genes, and R protein can also exert a negative regulation on the expression of *MIRNA* gene.

Virus-induced gene silencing (VIGS) is a common phenomenon during plant interactions with DNA or RNA viruses [28,29]. DCL-dependent production of virus-derived siRNAs is a marker of RNA-based immunity during plant-virus interactions (Figure 1) [30]. In *Arabidopsis*, 4 DCL proteins function differently in plant anti-RNA virus versus anti-DNA virus immunity. Among them, DCL2 and DCL4, the two major DCLs in defense against RNA viruses, function hierarchically in antiviral immunity [28,29]. In insects or fungi, only specific DCL proteins confer anti-viral activity [19]. The *Arabidopsis dcl2*/*dcl4* double mutant (resulting in the disappearance of 21/22 nt vsiRNAs) shows greater virus accumulation compared to wild-type plants [31]. DCL4-dependent secondary 21 nt vsiRNAs, rather than DCL2-dependent 22 nt vsiRNAs, seem to be more effective in plant anti-viral immunity during turnip crinkle virus (TCV) or cucumber mosaic virus (CMV) infection [32,33]. For DNA viruses (geminiviruses or pararetroviruses), 24 nt vsiRNAs enhance plant resistance through targeting the viral genome for DNA methylation modification, leading to TGS of viral genes [34,35]. This TGS regulation is carried out by the DCL3-dependent 24 nt siRNA-AGO4 complex [known as the RNA-directed DNA methylation (RdDM) machinery]. Loss of function of DCL3, AGO4 and double-stranded RNA binding protein 3 (DRB3) results in a significant increase in nuclear geminivirus minichromosome accumulation [34,36].

Despite primary vsiRNA synthesis, the amplification of secondary vsiRNAs by RDRs is also crucial for plant anti-viral immunity (Figure 1). There are 6 RDRs (RDR1~6) in *Arabidopsis*. RDR1, RDR2 and RDR6 play major roles in counteracting RNA viruses [37]. The RDR6-DCL4-DCL1 module is responsible for the biogenesis of transacting siRNAs (tasiRNAs) and natural antisense siRNAs (nat-siRNAs), which function similarly to miRNAs [38]. Systemic RNA silencing in *Arabidopsis* requires RDR1 and RDR6 for the amplification of CMV-derived sRNAs. CMV utilizes 2b proteins [viral suppressors of RNA silencing (VSR)] to suppress the slicer activity of plant AGO1 or AGO4 and RDR6-dependent RNA silencing [39]. A mutant form of CMV that does not express 2b proteins is nonpathogenic in wild-type and single-RDR-knockout *Arabidopsis* plants, but becomes highly virulent in *rdr1*/*6* double-knockout plants and *rdr1/2/6* triple-knockout plants [37]. Recently, two aminophospholipid-transporting ATPases 1 (ALA1 and ALA2) were identified through forward genetic screening of 2b-lacking CMV infection-sensitive mutants. ALA1 and ALA2 work together to enhance the RDR1 and RDR6-dependent synthesis of secondary vsiRNAs [40]. In addition, viruses can induce plant *RDR1* transcription, which is essential for the plant antiviral RNA silencing pathway [41]. Rice stripe virus (RSV) induces miR444 accumulation in rice to target three *MADS* genes that play repressive roles in *RDR1* transcription [42]. Compared to RNA viruses, RDR3, RDR4 and RDR5 might be responsible for the secondary vsiRNA synthesis of DNA viruses [35], as the *rdr1*/*rdr2*/*rdr6* triple mutant shows the same level of geminivirus accumulation as the wild-type [35]. Therefore, DCL protein-dependent vsiRNA and RDR-dependent secondary vsiRNA biogenesis in plants is highly flexible when a plant confronts different kinds of viruses.

RNA silencing-based immunity also exhibits cross-talk with R protein-mediated innate immunity during ETI [20]. In various plant species, *R* genes encoding NBS-LRR proteins (divided in two clusters of proteins, with a coiled-coil domain or TIR domain) that have been associated with ETI exhibit varying numbers [16]. Although R proteins confer robust resistance in contributing to the plant anti-pathogen interaction, unmanaged accumulation of R proteins results in autoimmunity, which inhibits plant growth and seriously negatively impacts agricultural production. It has been reported that several miRNAs directly target R genes for PTGS silencing [43,44,45]. Moreover, some R gene-targeting miRNAs are capable of inducing the production of RDR6-DCL3-dependent phased siRNAs (phasiRNAs) at cleaved R gene sites, resulting in trans-acting silencing (Figure 1). miR6019-miR6020-DCL4 mediated phasiRNA synthesis through the cleavage of *N* transcripts (encoding R proteins) in the absence of TMV infection is a fine-tuning process, showing how plants use miRNAs as a master regulator to achieve normal growth. Upon TMV infection, these two miRNAs were significantly decreased, increasing N protein-dependent immunity against TMV [43]. A subsequent study showed that the inverse relation between *N* transcript levels and miR6019/miR6020 levels dynamically changes during plant growth. Thus, in natural conditions, older tobacco plants show improved resistance against TMV infection compared to younger ones [45]. Interestingly, a recent study even showed that another *R* gene-encoding protein, SNC1, can translocate to the nucleus and repress the transcription of miRNA and phasiRNA loci, probably through the transcriptional corepressor TPR1. This repression indicated a potential SNC1-miRNA-phasiRNA module that reinforces plant immune responses upon virus infection [44]. Thus, miRNA-*R* gene-phasiRNAs is an important module for plant immunity against virus infection, but more effort should be focused on determining the conservation of this module upon infection by different virus species.

### 2.2. sRNAs in Plant-Bacteria Interaction

Recent reports have revealed that several specific miRNAs or siRNAs can be induced in plants during bacterial infection upon the perception of PAMPs or effectors [46,47]. For example, miR393 was significantly induced by flg22 in *Arabidopsis*, thereby increasing the silencing of auxin signaling receptor target genes and the host PTI response [48]. Deep sequencing data for AGO1-bound sRNAs induced by flg22 treatment indicated that upregulated miRNAs positively regulate plant immunity through the silencing of auxin receptor genes. Whether other PAMPs can trigger plant miRNA or siRNA accumulation is unknown. In another report, miR393b* induced by *Pseudomonas syringae* carrying the effector avrRpt2 conferred increased exocytosis of antimicrobial pathogenesis-related protein 1 (PR1) through AGO2-mediated silencing of the *MEMB12* gene [49]. Similarly, the endogenous phased, secondary siRNA (phasiRNA) nat-siRNAATGB2, which is DCL1 but not DCL4 dependent and can confer robust anti-bacterial resistance in plants, is significantly induced in *Arabidopsis* by avrRpt2 [50]. Interestingly, the effector avrRpt2, but not avrRpm1 or avrRps4, can induce 30–40 nt-long siRNAs (lsiRNAs) that are capable of silencing the resistance-related gene *AtRAP* [51]. Considering that the divergence of virulent or avirulent bacterial strains mainly depends on gain or loss of effectors [52], we assume that bacterially induced siRNA synthesis in plants is closely related to these different effectors.

### 2.3. sRNAs in Plant-Fungi and Plant-Oomycetes Interaction

Studies addressing sRNA synthesis induced by fungi or oomycetes were once rare because the contribution of these sRNAs to plant immunity was debated until recent years. 

Efforts to identify the RNA silencing suppressor activity of *Phytophthora* (an important model oomycete) effectors showed that two effectors, Phytophthora Suppressor of RNA Silencing 1 (PSR1) and PSR2, can suppress transgene-mediated *GFP* silencing in *GFP*-transgenic *N. benthamiana* by inhibiting the biogenesis of plant sRNAs [53]. Functional analysis of PSR1 indicated that its binding to the RNA helicase PSR1-Interacting Protein 1 (PINP1) impairs the biogenesis of both miRNA and siRNA, possibly through disassembly of dicing complexes (PSR1) [54]. A recent study demonstrated that miR161 (but not miR173)-mediated phasiRNA synthesis is crucial for *Arabidopsis* immunity upon *Phytophthora* invasion (42). However, this defense can be suppressed by the presence of PSR2, which has been shown to exhibit silencing suppressor activity through its interaction with the host DRB4 protein [55]. However, this mechanism is strain dependent, as no similar phenomenon has been observed in other *Phytophthora* strains (lacking the PSR2-encoding gene) with different host preferences [56]. 

*Verticillium dahliae* is a vascular invasive fungus [57]. A recent study indicated that the expression of 4 miRNAs was remarkably increased in *Arabidopsis* post-*Verticillium dahliae* infection. These 4 miRNAs target *ARF10*, *NAC1*, *PHV* and *ARF6*, in *Arabidopsis* [23]. Interestingly, this finding was similar to the interaction of *Arabidopsis* with *Pseudomonas syringae* infection, in which auxin signaling is involved (32). sRNA sequencing data from tomato plants infected by *Botrytis cinerea* (causal agent of gray mold) showed that induced phasiRNAs were most abundant; however, the biological functions of their corresponding target genes are still elusive [22]. Similarly, the accumulation of 51 miRNAs was upregulated in oilseed rape upon infection by *Sclerotinia sclerotiorum* (causing *Sclerotinia* stem rot). However, the functions of the target genes of these miRNAs are unknown [58]. In these cases, which effectors or PAMPs are responsible for the induction of sRNA biogenesis in plant is still elusive. Whether plants counteract the silencing suppressor activity of fungi or oomycete effectors is also not clear. These cases indicate that sRNAs are involved in modulating plant immunity against oomycetes and fungi, but more attention should be given to signal transduction in sRNA biosynthesis and the biological function of sRNAs targeting genes.

## 3. The Transportation of Trafficking sRNAs in Plants

In transgenic plants carrying hairpin RNAi constructs that target virulence-related genes of nematodes, fungi and parasitic plants, siRNAs are induced and enter pathogen cells to cause gene silencing. This silencing phenomenon is known as host-induced gene silencing (HIGS), which has been proven to be an ideal crop protection method for various pathogens [25,59,60,61,62,63,64]. For example, overexpression of siRNAs targeting the nematode virulence gene *16D10* in *Arabidopsis* can attenuate the invasion of nematodes [59]. Similarly, overexpression of siRNAs targeting the *Blumeria graminis* development gene *1,3-ß-glucanosyltransferase* (*GTF1*) or the effector-encoding genes *Avra10* and *Avrk1* in *Arabidopsis* can suppress the virulence of fungi [60]. *Arabidopsis* plants overexpressing siRNAs targeting *Botrytis cinerea DCL1/2* genes also display improved resistance [25]. In this case, *BcDCL1/2*-derived siRNAs translocated from host plants are detected in *Botrytis cinerea* cells. Similarly, overexpression of artificial sRNAs targeting the dodder *SHOOT MERISTEMLESS-Like* (*STM*) gene, which promotes cytokinin biosynthesis in the shoot apical meristem, can significantly attenuate the growth of dodder and improve host resistance in tobacco [61].

Interestingly, studies on the plant-*Verticillium*, plant-*Botrytis* (through extracellular vesicles mediated sRNAs transportation, we will discuss this phenomenon later) or *Cuscuta pentagona/Cuscuta campestris* (also known as dodder) interaction have indicated that natural HIGS is a plant native defense mechanism [65,66,67]. Through deep sequencing of total sRNAs from *Verticillium* hyphae recovered from infected cotton plants, Zhang et al. identified two cotton sRNAs, miR166 and miR159, that translocate into *Verticillium* cells. MiR166 and miR159 are specifically induced in cotton by *Verticillium* invasion, and translocated miR166 and miR159 can silence the virulence-related genes *Ca^2+^-dependent cysteine protease* (*Clp-1*) and *isotrichodermin C-15 hydroxylase* (*HiC-15*), respectively, to improve cotton resistance [65]. Surprisingly, MiR166 and miR159 can still be detected in mycelia after 20 d of culture, which indicates possible amplification of plant sRNAs in fungi. *Arabidopsis*-*Cuscuta pentagona* or *Cuscuta campestris* (also known as dodder) is a representative model for plant–parasite interactions. A recent report indicated that several 22 nt miRNAs from dodder were translocated in *Arabidopsis* and tobacco tissues to mediate defense-related mRNA degradation in host plants [67].

Other evidence has also suggested that fungal siRNAs targeting plant resistance-related genes can translocate into plant cells and attenuate plant immunity [24]. This phenomenon is known as pathogen-induced gene silencing (PIGS), which has been discovered in several cases of plant-pathogen interactions. For example, BcsiR3.1, BcsiR3.2 and BcsiR5 can translocate into *Arabidopsis* cells and silence the immunity-related genes *AtPRXIIF*, *AtMPK1/AtMPK2* and *AtWAK*, respectively, through a mechanism involving plant AGO1 [24]. Interestingly, BcsiR37, which silences eight *Arabidopsis* immunity-related genes, can be translocated into plant cells and cause plants to be susceptible to fungal infection [68]. Similarly, *Puccinia striiformis* microRNA-like RNA 1 (Pst-milR1) targeting wheat (*Triticum aestivum*) immunity-related gene *pathogenesis-related 2* (*PR2*) for full virulence is also mobile during fungal infection [69].

According to the above evidence related to HIGS, in natural HIGS and PIGS events, the mechanism of cross-kingdom RNAi is bidirectional during plant–fungus interaction [25] (Table 1). Despite these highly intriguing findings, the transportation mechanism of mobile sRNAs from plant to pathogen is still elusive. 

### 3.1. sRNA Transportation within Plants

The vascular system consisting of xylem and phloem is an essential and conserved structure in land plant species. Phloem contains three components: sieve elements [including plasmodesmata (PD) and callose], parenchyma cells and supportive cells [70]. Phloem is crucial for transporting small molecules (including water, ions or phytohormones) and large molecules (including mRNAs, sRNA and proteins) [71]. PD is the communicating channel for large molecule transportation between neighboring cells [72], which is well studied as a key component of mRNA transportation [73,74,75,76,77].

The translocation of sRNAs within plant tissues has long been viewed as a systemic signal. When tobacco leaves harboring a GFP transgene are infiltrated with *Agrobacterium tumefaciens* carrying a *GFP* reporter gene, *GFP* silencing can be observed in newly formed upper leaves [78]. This phenomenon indicated that siRNA may be transported through phloem. Later, PTGS mutation analysis showed that SDE1 (now known as RDR6) is responsible for transgene-mediated PTGS, but not virus-mediated PTGS [79]. The use of artificial siRNAs (based on *SUC2-SULi* and *SUC2-PDSi* constructs) targeting the *SULPHUR (SUL)* and *PHYTOENE DESATURASE* (*PDS*) genes in plants showed that these siRNAs can spread from companion cells to mesophyll cells and silence *SUL* and *PDS* gene expression [80]. The silencing of these genes relies on RDR6 and SDE3 (a putative RNA helicase) [81]. Moreover, the microRNA and heterochromatic silencing-related components RDR2 and NRPD1 (the largest subunit of plant-specific DNA-dependent RNA polymerase IV) were also found to be responsible for the intercellular transmission of RNA silencing in plant [82]. Thus, plant sRNAs are transported through phloem with the cooperation of the RNAi machinery.

With the exception of mobile siRNAs, few miRNAs have been reported to be translocated through phloem to regulate plant biological processes. Under phosphate (Pi) starvation, miR399 is induced and transported from shoots to roots through phloem to degrade the *PHO2* transcript, which encodes a critical component for the maintenance of Pi homeostasis, demonstrating that mobile miRNA is involved in the systemic control of detailed biological processes [83]. During legume-rhizobial interaction, tight control of symbiosis is required to balance plant growth and nodule numbers. Such regulation is achieved through shoot-to-root translocation of miR2111, which targets the symbiosis suppressor gene *TOO MUCH LOVE* (*TML*) for degradation to promote nodule formation [84]. Similarly, miR395 is translocated through phloem from shoots to roots and silences the *ATP sulfurylase 4* (*APS4*) gene, which is required for the maintenance of sulfate homeostasis, in *Brassica napus* roots under nutrient deficiency [85].

Plant mRNA transportation depends on assistance from RNA binding proteins (RBPs) in phloem, but not in a manner that involves passive diffusion [86]. Additionally, RBPs might protect mRNA from degradation during long-distance transportation within plant phloem [87]. Biochemical analysis of pumpkin phloem sap led to the characterization of *C. maxima* Phloem SMALL RNA BINDING PROTEIN1 (CmPSRP1) which is the only known RBP for sRNA transportation in plants. However, there is no ortholog of *CmPSRP1* in *Arabidopsis*. CmPSRP1 binds selectively to 25 nt ssRNA species. Microinjection studies indicated that PSRP1 mediates the cell-to-cell trafficking of 25 nt ssRNAs through PD in the phloem [88]. CmPSRP1–sRNPC (an sRNA ribonucleoprotein complex) functions in the systemic delivery of phloem-mobile sRNAs. This delivery depends on the phosphorylation of PSRP1 by the phloem-localized protein kinase PSRPK1. Dephosphorylation of PSRP1 might contribute to ssRNA release in target cells [89]. However, whether PSRP1 can transport miRNA or phasiRNA is not understood. It is also unknown whether any pathogen-induced RBP exists in plants.

Since many components involved in sRNA transportation within plants are also key players in plant-antiviral immunity, this transportation mechanism could provide guidance in studying the cross-kingdom transportation of plant sRNAs.

### 3.2. Cross-Kingdom Transportation of Plant sRNAs through Extracellular Vesicles (EVs)

Cell–cell communication in plants or animals allows the coordination of cell functions during growth and environmental inhabitation (including the response to biotic or abiotic stress) [90]. In mammals, EVs containing mRNA, miRNA, extracellular miRNA, noncoding RNA and DNA that can be exchanged between cells represent a crucial pathway in growth and stress responses [91]. Mammalian EVs can be classified as exosomes, shedding vesicles or apoptotic bodies. Exosomes contain proteins that are important for exosome membrane transport and fusion (marker proteins such as RAB GTPases or annexins), cytoskeletal proteins, adhesion molecules and tetraspanin family proteins (marker proteins such as CD81, CD82 and CD63) and RAB proteins (marker proteins such as RAB11, RAB27 and RAB35), which are involved in regulating exosome secretion [90]. Exosomes are intraluminal vesicles (ILVs) assembled inside multivesicular bodies (MVBs) that are responsible for releasing exosomes through their fusion with the plasma membrane. The fusion of MVBs with the plasma membrane is possibly aided by a complex of SNARE proteins [92]. Exosomes in animals are released constitutively, however their secretion can be increased in the cellular response to immune activation [90]. In mammals, immune synapses formed at the T cell–antigen presenting cell (APC) interface are important in T cell activation and the delivery of effector molecules such as cytokines and lytic granules [90]. A recent study showed that the exosomes of T, B and dendritic immune cells contain miRNAs that are exchanged during cognate immune interactions in synapse formation. When these miRNAs are transferred to recipient cells, they can modulate gene expression, which supports a mechanism of cell–cell communication involving the intercellular transfer of miRNAs by exosomes during immune synapsis [93].

In plant systems, EVs comprise two major classes: microvesicles and exosomes (with markers such as tetraspanin 8/9, TET8/9, and *PENETRATION 1*, *PEN1*), based on their diameter [94]. Plant exosome release also relies on MVBs (with markers such as Rab5-like GTPase, ARA6), which should properly fuse with the cell membrane [95,96,97]. Because of the requirement of host tissue colonization, a parasitic plant haustorium structure is often formed adjacent to the host phloem system. Thus, the EV-mediated immune response might occur within such an area [98,99]. Recently, by combining transmission electron microscopy (TEM) tomography and three-dimensional (3D) reconstruction technologies, researchers identified a structure between plant and fungal cells known as the peri-arbuscular space. The plasma membrane of this space is surrounded by MVBs [100,101], which implies the possible release of EVs within this space. *Arabidopsis*-*Blumeria graminis* f.sp. *hordei* (*Bgh*) (a powdery mildew fungus) is an ideal model system for nonhost or host interaction studies. This system showed that constitutive formation of papilla and encasement results in changes in host and nonhost interactions [102]. Reverse genetic screening of this model system identified three genes, *PEN1*, *PEN2* and *PEN3*, as key players in mediating plant innate immunity against *Bgh* through an exocytic pathway [102,103,104]. Loss function of PEN2 and PEN3 can cause host invasion of *Bgh* (a shift from nonhost to host interaction) through defects in the host transcytosis of glucosinolate derivatives (a toxin compound involved in pathogen growth). Loss of function of PEN1 can cause host invasion of *Bgh* through defect in exosome release.

**Figure 2:**
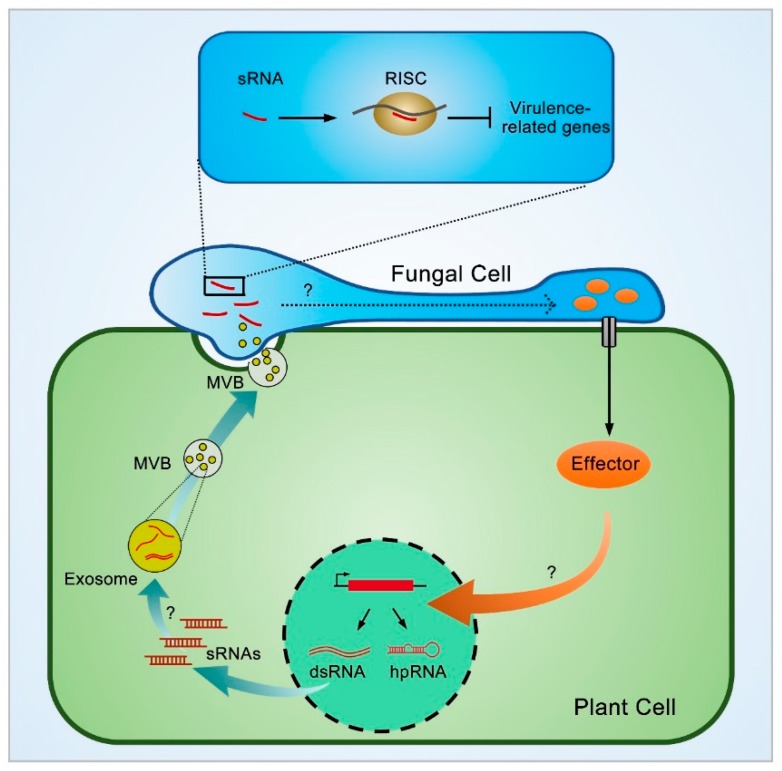
A working model of trafficking sRNA-dependent regulation of plant immunity during plant–fungus interaction. DsRNA and folded hairpin (hp) RNA are firstly produced from exogenous transgenes or endogenous host genes, and then sRNAs are processed by Dicer from the above dsRNA precursors. Through an unknown mechanism, specific sRNAs are loaded into exosomes with unknown isoform. sRNA-containing exosomes are assembled into multivesicular bodies (MVB) and translocated into fungal cell through transcytosis-mediating exosome release. Then plant trafficking sRNAs cooperate with fungal RISC to silence virulence related genes and improve plant resistance. Meanwhile, fungi can secret effectors into plant cells to interfere with host RNA silencing pathway, thereby disrupting host immunity. MVB, multivesicular bodies, RISC, RNA-induced silencing complex. Solid arrow indicates the direction of the working model. The dashed arrow indicates the possible mechanisms.

Studies concerning improving the isolation method for plant EVs successfully demonstrated that proteins and sRNAs inside EVs are required for pathogen resistance (Figure 2) [105,106]. For example, experiments involving EVs from sunflower seedlings showed that the proteins inside EVs can be absorbed by fungi and inhibit fungal growth [106]. In the *Arabidopsis*-*Phytophthora* pathosystem mentioned previously, researchers have also detected phasiRNAs derived from the EVs of infected *Arabidopsis* leaves. These phasiRNAs can target *Phytophthora* virulence-related genes and result in attenuated invasion (Figure 2) [55]. In the *Arabidopsis-Botrytis cinerea* pathosystem, tasiRNAs and heterochromatic siRNAs are transported into fungi by EVs, which results in improved plant resistance. In this case, it has been shown that the transportation of these mobile sRNAs in exosomes does not occur through concentration-dependent diffusion but possibly takes place through a selective process. sRNA profiling of the total RNAs of *Arabidopsis* leaves and EVs showed a clear selection bias in transferred sRNAs in EVs. Only 3 siRNAs (TAS1c-siR483, TAS2-siR453 and IGN-siR1) were proven to be absorbed and to function in fungal cells (Figure 2) [66]. In mammals, this phenomenon is also similar to exosome-derived-sRNA sequencing results indicating that certain miRNA populations are selectively assembled into exosomes [93]. However, the selection criteria are not clear. Some mobile mRNAs have been found to harbor a tRNA-like sequence (TLS) originating from TMV or BMV [107]. Deletion of the TLS sabotages the mobility of these mRNAs but not their transcription [108]. Whether a TLS is present in the precursor of plant mobile sRNAs is unknown.

However, how EVs establish connections with fungus or parasite cells and how sRNAs are transported into fungal cells remains to be elucidated. EVs might be responsible for systemic RNA transportation in animals [109], and we speculate that this is also true in plants. Additionally, whether a pathogen might hijack plant EVs to achieve successful infection is also unknown. Moreover, the poor understanding of plant EVs means that it is unknown how EVs can travel across the cell plasma membrane and cell wall. The answer might be found in a future study concerning the biogenesis of MVBs, as Cai et al. showed the partial colocalization of an MVB marker (ARA6) and exosome marker (TET8) [66]. In brief, EVs have been shown to be involved in the modulation of plant immunity, but how EV biogenesis and release contribute to cross-kingdom RNA silencing during pathogen invasion needs to be illustrated in the future studies. 

## 4. Plant Mobile sRNA-Mediated Gene Silencing in Pathogens

### 4.1. The Working Mechanism of Plant sRNA in Fungi

The plant–fungus interaction is an ideal system for dissecting the molecular mechanism of plant mobile sRNA-mediated gene silencing in pathogen cells (Figure 2). Unlike the situation in plants and animals, RNAi in fungi is well studied not only in fission yeast and *Neurospora crassa* (*N. crassa*) but not also in invasive plant fungi such as *Magnaporthe oryzae* (*M*. *oryzae*), *Botrytis* and *Fusarium graminearum* (*F. graminearum*). RNAi is achieved mainly through two pathways known as quelling (repetitive transgene-induced gene silencing) and meiotic silencing (unpaired DNA induced-gene silencing) in *N. crassa* [110]. Genetic studies of the key components in these two pathways demonstrated similar working mechanisms despite the difference in the initiation of dsRNA precursors [110]. This rule has also been found to apply in limited RNAi studies in other plant-invasive fungi such as *Aspergillus nidulans*, *Cryphonectria parasitica* and *M*. *oryzae* [110]. Plant mobile sRNA might use the RNAi machinery of fungi or other pathogens to silence virulence-related genes; however, the answers to questions concerning the functional molecular form of plant mobile sRNA in fungal cells and the existence of a plant mobile sRNA-fungus AGO complex are far from clear.

To date, no evidence has revealed the determinate molecular form of mobile sRNA (long dsRNA or ssRNA) within plant or fungal cells. The molecular form of plant endogenous sRNAs derived from heterochromatin regions or *TAS* mRNA precursors transported by EVs is also elusive. In plant grafting assay, even long dsRNA precursors in roots can be transported and mediate RNA silencing in ectopic shoots, and NRPD1a, RDR2 and DCL3 are required in this process [111]. In vitro synthesis of *YFP* fluorescein-labeled RNAs absorbed by *Botrytis* cells indicated that both sRNA duplexes and long double-stranded RNAs are capable of triggering *DCL1* and *DCL2* silencing [25]. The possibility that fungal DCLs might process plant mobile sRNA into a functional molecular form has only been confirmed in the barley–*F. graminearum* interaction. DCL1 is not required for successful barley leaf infection by *F. graminearum*, but it is crucial for fungal gene silencing mediated by artificial dsRNA absorption. The sRNA sequencing of *dcl1* and wild-type strains grown in axenic medium with artificial dsRNA further showed abundance of sRNAs derived from artificial dsRNAs only in the wild-type strain and not in the *dcl1* strain [112].

When plant mobile sRNAs are translocated into fungus cells, it is important to understand whether these sRNAs are directly loaded into fungus AGO to form a silencing complex. Thus, it is important to understand the characteristics of functional sRNAs (length and 5′ terminal nucleotide bias) in fungal AGO proteins. The systemic analysis of *Mo*AGO1-3 showed that all three AGO proteins are not required for the appropriate growth rate, germination, appressorium formation and infectivity. However, AGO1 and AGO3 are indispensable in gene silencing and resistance to mycoviruses. The sRNAs of the three AGO proteins sequenced via AGO-IP assays are typically 19-20 nt with a 5′-U bias, similar to the AGO1 protein in *Arabidopsis* [113]. It is possible that plant mobile sRNAs processed by the fungal DCL protein with a 5′-U bias could be loaded into a fungal AGO protein for gene silencing. However, there is little evidence supporting this hypothesis thus far. The analysis of sRNA abundance derived from artificial dsRNAs in fungi will be a good first step in a future study.

Taken together, the functional molecular form of plant mobile sRNAs in fungal cells and the existence of plant mobile sRNA-fungus AGO complexes are important hints for elucidating the functional mechanism of plant sRNAs in fungi. To reveal the detailed mechanism underlying trafficking sRNA-mediated cross-kingdom silencing, an ideal genetic screening system is desperately needed for studies on plant-pathogen interactions.

### 4.2. Application of Trafficking sRNAs in Improving Plant Resistance

Two widely accepted goals in anti-pathogen crop breeding are to reduce insecticide or germicide use and achieve broader-spectrum pathogen resistance. The arms race between plants and pathogens allows rapid evolution of pathogen virulence, which usually causes substantial economic loss worldwide. Recently, the application of the spray-induced gene silencing (SIGS) approach (spraying with artificial dsRNAs targeting pathogen virulence-related genes) to control fungi and pest populations in plants was introduced. SIGS is different from HIGS because no transgenic methods are required for SIGS. Despite the higher cost of dsRNA synthesis and the shorter effective period, SIGS shows that the future use of siRNA as a biopesticide is promising compared to the long period required for anti-pathogen crop breeding to achieve stable transgenic lines [114]. However, the quantity of sRNAs that is sufficient for triggering gene silencing in fungi is not clear. One study indicated that spraying only 20 μg of synthetic sRNAs (including dsRNA and siRNA) on the surface of an individual plant could inhibit the invasion and growth of *Botrytis* [25]. Spraying one barley leaf (local) with 10 μg of artificial dsRNA targeting three *F. graminearum* cytochrome P450 lanosterol C-14α-demethylases (required for the biosynthesis of fungal ergosterol) inhibits fungal growth in both directly sprayed (local) and nonsprayed (distal) leaves [112]. However, whether these quantities are also effective in other plant fungi or parasites is unknown. Interestingly, dsRNA loaded on layered double hydroxide (LDH) clay nanosheets shows sustained release and stable storage characteristics and is effective in inhibiting CMV [115]. Combining dsRNA with nanomaterials is now recognized as promising approach in crop breeding applications. Moreover, attention still needs to be focused on how much siRNA transportation efficiency can be improved. Spraying is obviously an easier approach in practice, but recent studies involving human exosome-mediated therapy [116] might provide another alternative. This approach involves the injection of exosomes with proteins or RNAs of interest to form the “cargo”. This cargo is then injected back into mammalian cells, which works effectively in cancer therapy [116]. If the introduction of desired siRNAs to plant exosomes can be successfully carried out, reduction of the number of RNAs, a lower chance of degradation and a higher silencing efficiency might be achieved. Once mobile sRNAs are translocated to guide specific gene silencing in pathogen cells, the remaining question will be whether this mobile sRNA-mediated silencing could be inherited in transgenerational manner. Thus, future efforts toward improving SIGS efficiency and developing a new sRNA transportation medium might be key in crop anti-pathogen breeding.

## 5. Concluding Remarks and Future Perspectives

The finding that plant mobile sRNAs can trigger cross-kingdom silencing in pathogen cells is of great interest and provides a novel layer of regulation for the interplay between plant hosts and pathogens (Figure 2). There are three components of this process: sRNA biogenesis in host plants, sRNA transportation from host plants to pathogen cells and sRNA-mediated gene silencing in pathogen cells. Knowledge concerning the transportation and molecular mechanisms of mobile sRNA-mediated gene silencing in pathogen cells is scarce; therefore, we think that the following question should be addressed in future work:

(1) What are the common molecular components (effectors or small molecular compounds) involved in pathogen-induced sRNA biogenesis in plants?
(2) Is any RBP involved in transporting plant sRNAs into pathogen cells?
(3) How are trafficking sRNAs selected? In other words, what is the selective criterion for sRNA translocation in EVs?
(4) How do EVs establish connections with fungus or parasite cells? How are sRNAs released from plant cells and absorbed by pathogen cells?
(5) What is the molecular form of plant mobile sRNAs in pathogen cells?
(6) Which components are required for plant mobile sRNA-mediated gene silencing in pathogen cells?

## Figures and Tables

**Table 1:** Cross-kingdom trafficking sRNAs in plant-pathogen interaction.

sRNAs	Origination	Interaction	Target Genes	Reference
siR1310	*Arabidopsis*	*Arabidopsis-Phytophthora*	fungal virulence gene	[55]
miR166	*Gossypium*	*Gossypium-Verticillium*	fungal virulence gene	[65]
miR159	*Gossypium*	*Gossypium-Verticillium*	fungal virulence gene	[65]
TAS1c-siR483	*Arabidopsis*	*Arabidopsis-Botrytis*	fungal virulence gene	[66]
TAS2-siR453	*Arabidopsis*	*Arabidopsis-Botrytis*	fungal virulence gene	[66]
IGN-siR1	*Arabidopsis*	*Arabidopsis-Botrytis*	Unknown	[66]
BcsiR3.1	*Botrytis*	*Arabidopsis-Botrytis*	host *PRXIIF* gene	[24]
BcsiR3.2	*Botrytis*	*Arabidopsis-Botrytis*	host *MPK1*/*MPK2* genes	[24]
BcsiR5	*Botrytis*	*Arabidopsis-Botrytis*	host *WAK* gene	[24]
BcsiR37	*Botrytis*	*Arabidopsis-Botrytis*	Eight host genes	[68]
Pst-milR1	*Puccinia*	*Triticum-Puccinia*	*PR2* gene	[69]
miR12495	*Cuscuta*	*Arabidopsis-Cuscuta*	host *BIK1* gene	[67]
miR12497a	*Cuscuta*	*Arabidopsis-Cuscuta*	host *TIR1/AFB2/AFB3* genes	[67]
miR12463b	*Cuscuta*	*Arabidopsis-Cuscuta*	host *BIK1* gene	[67]
miR12480	*Cuscuta*	*Arabidopsis-Cuscuta*	host *SEOR1* gene	[67]
